# Prenatal inflammation-induced NF-κB dyshomeostasis contributes to renin-angiotensin system over-activity resulting in prenatally programmed hypertension in offspring

**DOI:** 10.1038/srep21692

**Published:** 2016-02-15

**Authors:** Youcai Deng, Yafei Deng, Xiaoyan He, Jianhong Chu, Jianzhi Zhou, Qi Zhang, Wei Guo, Pei Huang, Xiao Guan, Yuan Tang, Yanling Wei, Shanyu Zhao, Xingxing Zhang, Chiming Wei, Michael Namaka, Ping Yi, Jianhua Yu, Xiaohui Li

**Affiliations:** 1 Institute of Materia Medica, College of Pharmacy, Third Military Medical University, Chongqing, China; 2 Division of Hematology, Department of Internal Medicine, The Ohio State University, Columbus, Ohio, USA; 3 Jiangjin District Central Hospital, Chongqing, China; 4 Suzhou Institute of Blood and Marrow Transplantation, Soochow University, Suzhou, China; 5 Department of Pharmacy, Hospital 159 of PLA, Zhumadian, Henan, China; 6 Chongqing Center for Biomedicine and Medical Equipment, Chongqing Academy of Science and Technology, Chongqing, China; 7 Colleges of Pharmacy and Medicine, University of Manitoba, Apotex Center 750, McDermot Avenue, Winnipeg, MB, Canada; 8 Joint Laboratory of Biological Psychiatry Between Shantou University Medical College and the College of Medicine University of Manitoba, Canada; 9 Department of Obstetrics and Gynecology, Daping Hospital, Third Military Medical University, Chongqing, China

## Abstract

Studies involving the use of prenatally programmed hypertension have been shown to potentially contribute to prevention of essential hypertension (EH). Our previous research has demonstrated that prenatal inflammatory stimulation leads to offspring’s aortic dysfunction and hypertension in pregnant Sprague-Dawley rats challenged with lipopolysaccharide (LPS). The present study found that prenatal LPS exposure led to NF-κB dyshomeostasis from fetus to adult, which was characterized by PI3K-Akt activation mediated degradation of IκBα protein and impaired NF-κB self-negative feedback loop mediated less newly synthesis of *IκBα* mRNA in thoracic aortas (*gestational day 20, postnatal week 7 and 16*). Prenatal or postnatal exposure of the IκBα degradation inhibitor, pyrollidine dithiocarbamate, effectively blocked NF-κB activation, endothelium dysfunction, and renin-angiotensin system (RAS) over-activity in thoracic aortas, resulting in reduced blood pressure in offspring that received prenatal exposure to LPS. Surprisingly, NF-κB dyshomeostasis and RAS over-activity were only found in thoracic aortas but not in superior mesenteric arteries. Collectively, our data demonstrate that the early life NF-κB dyshomeostasis induced by prenatal inflammatory exposure plays an essential role in the development of EH through triggering RAS over-activity. We conclude that early life NF-κB dyshomeostasis is a key predictor of EH, and thus, NF-κB inhibition represents an effective interventional strategy for EH prevention.

The incidence and prevalence of cardiovascular disease continues to rise warranting it has the leading cause of death. About 17.3 million people have died per year, and it is expected that the number of deaths will continue rise to over 23.6 million by 2030^1^,^2^. Interestingly, the number of hypertension patients have been predicted to be between 1.54–1.58 billion by year 2025, representing one of the most important public-health challenges worldwide^3^. However, current available treatment approaches fail to halt this aspect of cardiovascular disease. This issue is further complicated by the fact that over 90% of hypertension cases have an unknown origin and are therefore classified as essential hypertension (EH)^4^.

Epidemiological studies suggest that the hypertensive manifestations in adults are associated with environmental factors introduced to the developing fetus during the prenatal period. As such, this type of hypertension is more commonly known as prenatally programmed hypertension (PPH)^5^. Prenatal inflammatory exposure to infection^6^, hepatitis^7^, and arthritis^8^ are some of the most common events that occurs in pregnant women. Prenatal exposure to these types of inflammatory stimuli are often associated with lower birth weight, preterm births, that suffer from PPH^9^,^10^,^11^. Despite great improvements in maternal health care to minimize exposure to infection during pregnancy, bacterial vaginitis represents a major cause that might associated with the development of PPH^12^,^13^. As a result of the literature that supports the development of PPH due to prenatal exposure to an inflammatory stimulus, we hypothesized that prenatal inflammatory stimulation is important in underlying pathogenesis of EH. As such, we have a well-established animal model of PPH that involves the exposure of pregnant Sprague-Dawley (SD) rats to an inflammatory stimulus induced by lipopolysaccharide (LPS), the main component of gram-negative bacteria cellular wall^14^, during the secondary trimester^15^,^16^,^17^,^18^. Interestingly, prenatal exposure to LPS was not associated with any lower birth weight or size of the pups and did not alter the sex ratio of the litter.

The conduit artery plays a dominant role in cushioning. Cushioning has the ability to dampen the pressure oscillations that result from intermittent ventricular ejection^19^. Aortic dysfunction leads to hypertension, which in turn contributes to the elevated morbidity and mortality associated with the known complications of cardiovascular disease^20^,^21^. A large longitudinal study of the general population recently has shown that aortic dysfunction precedes the elevation in systolic blood pressure that leads to the diagnosis of hypertension^22^. At present, there is extensive information that identifies the possible mechanisms that cause dysregulation of aortic function and subsequent elevation in systolic blood pressure. However, our research identifies an additional cause of aortic dysfunction in the offspring that received prenatal exposure to an inflammatory stimulus. Our results confirm the damage to the aortic microstructure in neonatal animals with increased medial thickness and higher contractility at the postnatal age of 12 weeks^18^,^23^. As such, our research demonstrates a direct role of prenatal inflammatory stimulation on aortic dysfunction, independent of increased blood pressure. However, the detailed mechanisms for aortic dysfunction are still largely unknown.

Almost all danger-sensing receptors of the innate and adaptive immune systems can activate NF-κB signaling to mediate effector function. NF-κB and IκB proteins form an integrated network in regulating NF-κB activation. NF-κB subunits, such as the transactivation domain–containing RelA (p65) and the binding to inhibitory IκB proteins, stay within the cytoplasm of mammalian cells in a form of various homodimers and heterotrimers in order to remain inactive during the resting state^24^. However, when cells are triggered by various inflammatory stimuli, the upstream I-κ kinase (IKK) complex becomes activated to phosphorylate IκBs, and consequently targets IκBs to proteasome degradation and frees NF-κB dimers to translocate to nucleus, where the dimers promote transactivation of various cytokines involved in the induction of an inflammatory response. These inflammatory responses can be rapidly reversed by newly synergized IκBα or maintained by newly synergized IκBβ^24^. In this regard, we previously found a higher inflammatory response in whole embryos after prenatal LPS stimulation^25^ and NF-κB activation in thoracic aortas obtained from animals that received prenatal LPS exposure at the postnatal age of 12 weeks^18^. However, the roles and mechanisms of NF-κB signaling abnormality in the conduit artery before the hypertensive manifestation remain largely unknown.

Vascular dysfunction is highly related to vascular renin-angiotensin system (RAS) over-activity^26^. Endothelium dysfunction (classically defined as impairment in its vasodilatory capacity), includes a decrease in endothelial nitric oxide synthase (eNOS) bioactivity and a lack of nitric oxide (NO)^27^. The RAS family mainly contains angiotensinogen, renin, angiotensin I, angiotensin-converting enzyme (ACE), ACE2, angiotensin II (Ang II), Ang II type 1 receptor (AT1R), and Ang II type 2 receptor (AT2R). Compared to the circulation RAS, the local tissue RAS components are known to participate in the chronic cardiovascular events such as secondary structural damage thereby contributing more to the pathogenesis of hypertension^28^. Previous findings in spontaneous hypertensive rats had shown that the overexpression of ACE was only found in conduit but not resistance artery^29^. In light of the critical role of RAS dysfunction in the development of hypertension and our previous finding of increased AngII and AT1R in thoracic aortas of offspring with prenatal exposure to LPS^30^, we decided to explore the specific mechanisms responsible for RAS dysfuncton and its relationship with NF-κB activation in vascular tissue prior to the developmental onset of hypertension.

In the current study, we determined the changes of the NF-κB signaling pathway in both thoracic aortas and superior mesenteric arteries in our animal model of PPH. Based on our experience with this model, we hypothesized that early life and lifelong aortic NF-κB dyshomeostasis leads to RAS re-programming, which is associated with conduit artery dysfunction and development of hypertension (Fig. 1). To test this hypothesis, we evaluated the mRNA and protein expression of the NF-κB signaling components in thoracic aortas of inflammatory-induced fetal and adult PPH rats. In addition, we also used a specific IκBα degradation inhibitor, called pyrollidine dithiocarbamate (PDTC)^31^, which selectively prevents NF-κB activation^32^, to inhibit NF-κB activity during pregnancy or in adult animals. During this interventional treatment, we recorded the blood pressure, RAS activity and endothelium function in the offspring’s thoracic aortas.

## Results

### Prenatal inflammatory exposure disrupts offspring’s NF-κB signal homeostasis characterized by lower level of IκBα expression in thoracic aortas at early life predominantly through PI3K-Akt activation and impaired NF-κB self-negative feedback loop

**Figure 2:**
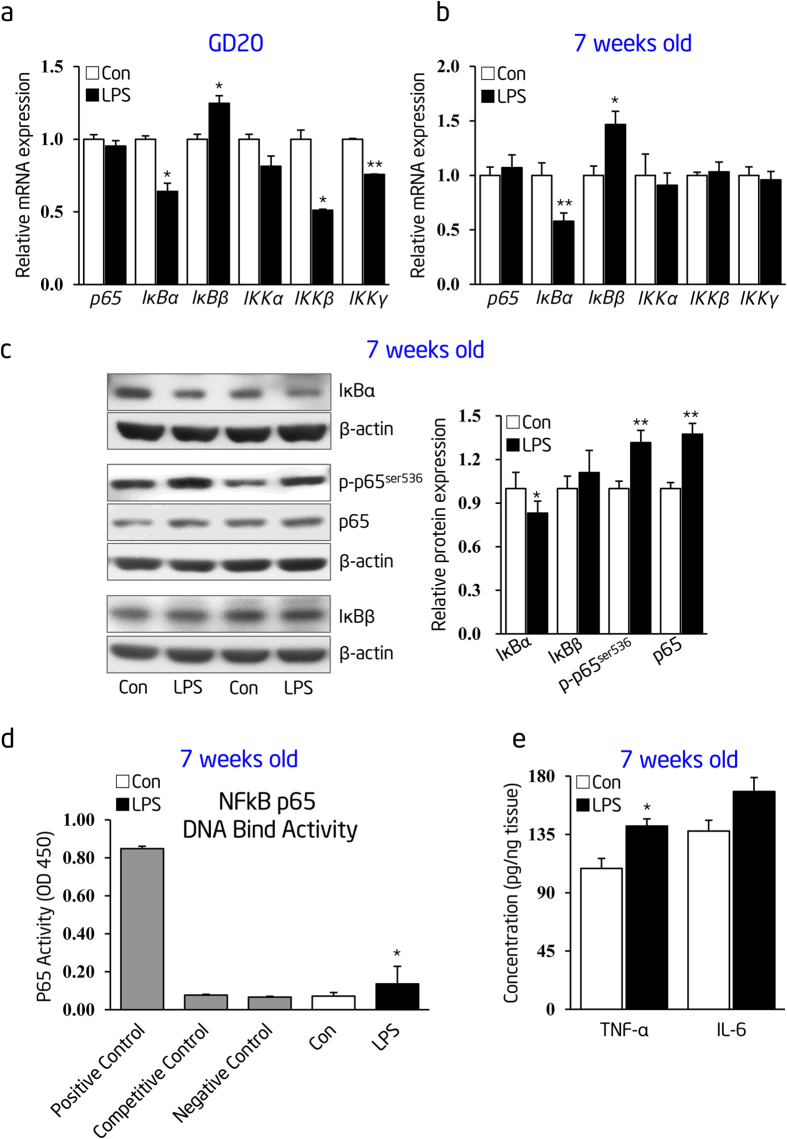
Prenatal LPS stimulation leads to early life thoracic aortic NF-κB dyshomeostasis. (**a**) and (**b**) Thoracic aortic *p65*, *IκBα*, *IκBβ*, *IKKα*, *IKKβ* and *IKKγ* mRNA expression were determined by real-time RT-PCR at gestational day 20 (**a**) and postnatal week 7 (**b**) (n = 6 offspring in each group). (**c**) IκBα, IκBβ, phosphorylated (p)-p65 and total p65 protein expression were determined by immunoblotting in thoracic aortas of offspring at postnatal week 7. β-actin was taken as internal control. Representative plots of 2 from 8 offspring in each group (left panel) and statistical data of relative densitometry, normalized by β-actin (right panel), were shown. (**d**) NF-κB p65 DNA binding activity in thoracic aortas of 7-week-old offspring were determined by a NF-κB EZ-TFA transcription factor assay colorimetric kit. Positive, competitive, and negative controls were carried out using TNF-α-treated Hela whole cell extract, unlabeled competitor oligonucleotide containing the identical consensus sequence as the capture probe in other samples, and without any capture probe, respectively (n = 6 offspring in each group). (**e**) Expression levels of TNF-α and IL-6 in the thoracic aortas of 7-week-old offspring were measured by ELISA (n = 8 offspring in each group). Con indicates prenatal vehicle control; LPS, prenatal LPS stimulation. Error bars represent S.D. *p < 0.05 and **p < 0.01, LPS versus Con.

Prior to the onset of hypertension, we observed aortic dysfunction with unknown mechanisms^18^. NF-κB signal is the main downstream pathway after the inflammation challenge. To determine the role of NF-κB signal in prenatal inflammatory-induced conduit artery dysfunction, we firstly assessed the mRNA and protein levels of NF-κB family components in the thoracic aorta of prenatal LPS-stimulated offspring at gestational day (GD) 20 or postnatal week 7. By real-time RT-PCR analysis, we observed that prenatal LPS stimulation significantly decreased *IκBα* but increased *IκBβ* mRNA expression in thoracic aortas of offspring at both GD 20 (Fig. 2a) and postnatal week 7 (Fig. 2b), as compared to control animals. In addition, prenatal LPS exposure significantly decreased the mRNA expression levels of *IKKβ* and *IKKγ* in offspring’s thoracic aortas at GD 20 (Fig. 2a); however, there was no significant change at postnatal week 7 (Fig. 2b). Immunoblotting further validated the decreased IκBα, increased phosphorylated (p)-p65 and total p65 protein levels in thoracic aortas of prenatal LPS-stimulated offspring at postnatal week 7, as compared with those in the control group (Fig. 2c). To find more direct evidences of NF-κB activation in the thoracic aorta of prenatal inflammation-induced offspring, we evaluated the NF-κB p65 DNA binding activity using a non-radioactive universal EZ-TFA Transcription Factor Assay kit. This kit combines the principle of the electrophoretic mobility shift assay (EMSA) with the 96-well based enzyme-linked immunoabsorbent assay (ELISA) instead of the conventional EMSA. Our data showed that p65 DNA binding activity in thoracic aortas of prenatal LPS-stimulated offspring was significantly higher than that in the control offspring (Fig. 2d). We also observed a significantly higher level of tumor necrosis factor-α (TNF-α), a downstream target of NF-κB, in thoracic aortas of prenatal LPS-stimulated offspring at the age of 7 weeks (Fig. 2e). These data indicate a predisposition of early life NF-κB activation, characterized by the low expression of IκBα at both the mRNA and the protein levels, in the conduit artery of prenatal inflammation-induced offspring.

**Figure 3:**
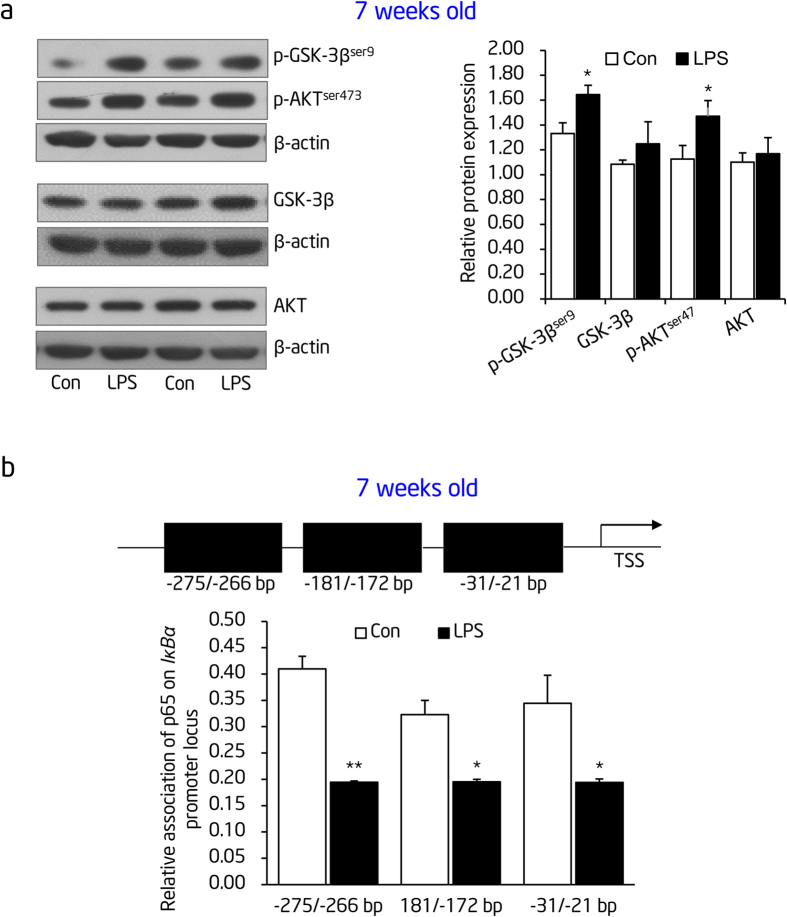
PI3K-Akt activation and impaired NF-κB self-negative feedback loop on *IκBα* promoter are implicated in reduced IκBα expression in thoracic aortas of offspring from prenatal LPS stimulation. (**a**) Expression of Akt, GSK-3β, phosphorylated (p)-Akt^ser473^ and p-GSK-3β^ser9^ at the protein level was determined by immunoblotting in thoracic aortas of offspring at postnatal week 7. β-actin was taken as internal control. Representative plots of 2 from 8 offspring in each group (left panel) and statistical data of relative densitometry, normalized by β-actin (right panel), were shown. (**b**) Schematic structure of the *IκBα* (*Nfkbia*) promoter with putative NF-κB-binding sites (top panel), and ChIP analysis of NF-κB (p65) binding to the *IκBα* promoter in thoracic aortas at the age of 7 weeks old (bottom panel) (n = 5 offspring in each group). Con indicates prenatal vehicle control; LPS, prenatal LPS stimulation. Error bars represent S.D. *p < 0.05 and **p < 0.01, LPS versus Con.

Interestingly, the mRNA expression of *IKK*s, which implicates in IκBα phosphorylation and degradation, did not significantly change at postnatal week 7, indicating that mechanisms upstream of IKK or other signal pathways contributed to the down-regulation of IκBα. Previous findings reported that PI3K-Akt activation leads to IκBα degradation through IKK-dependent^33^ and -independent pathway^34^,^35^. Our current finding of increased phosphorylated IκBα in thoracic aortas of offspring from prenatal exposure to LPS, promoted us firstly to explore whether there exists an activation of PI3K-Akt in thoracic aortas of offspring from prenatal exposure to LPS. As expected, phosphorylated Akt^ser473^ and its downstream target phosphorylated GSK-3β^ser9^ were significantly increased in thoracic aortas of offspring that received prenatal exposure to LPS at the age of 7 weeks (Fig 3a). To further address the mechanism by which *IκBα* mRNA expression was downregulated, we determined the binding ability of NF-κB (p65) to the promoter of *IκBα* (*NFKBIA*) because previous reports found that NF-κB can bind to multiple sites within the *IκBα* promoter thereby promoting its transcription in the recovery stage as a negative feedback loop after NF-κB activation^36^. Our chromatin immunoprecipitation (ChIP) assays found that decreased binding of p65 appeared on all three potential binding sites of *IκBα* promoter (−275/−266 bp, −181/−172 bp and −31/−21 bp) in thoracic aortas of offspring that received prenatal exposure to LPS (Fig. 3b). This tendency was also the same in offspring from prenatal exposure to LPS at the age of 16 weeks (Data not shown).

**Figure 4:**
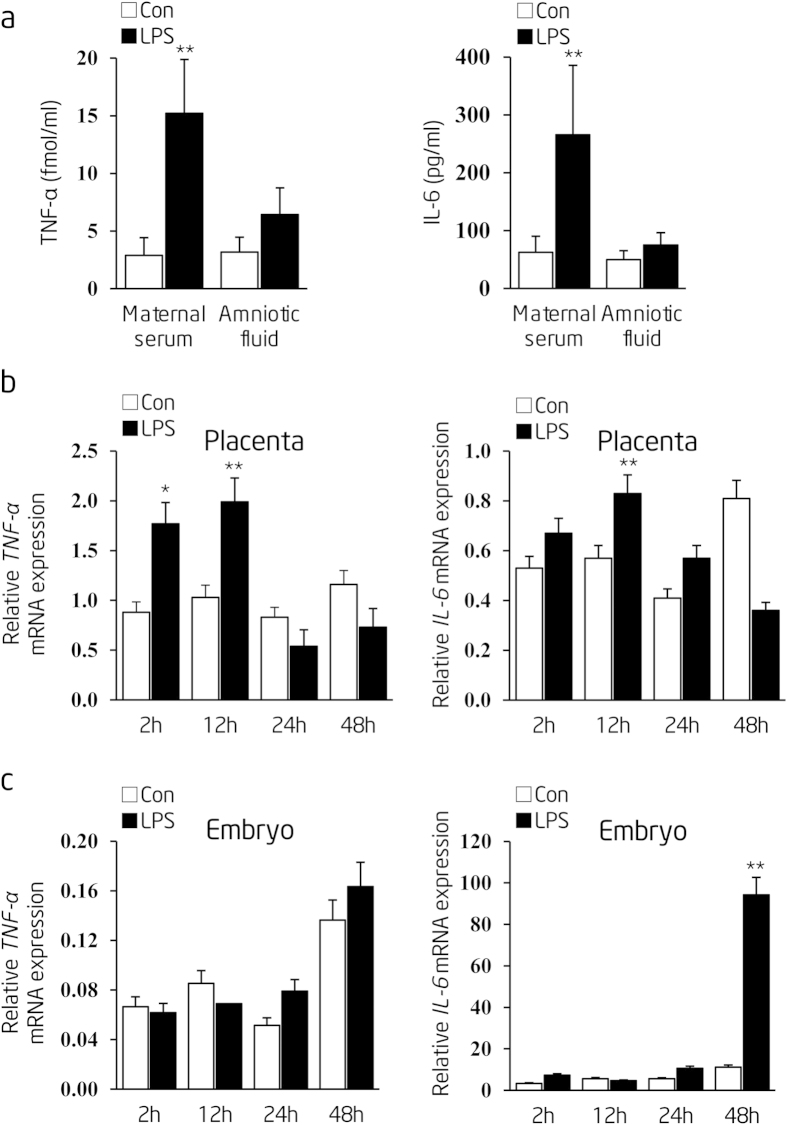
Prenatal LPS stimulation leads to fetal pro-inflammatory factors overexpression in an indirect manner. (**a**) TNF-α and IL-6 levels in maternal serum and amniotic fluid were measured by radioimmunoassay after 2 h of the last LPS i.p injection (n = 3 pregnant rats in each group). (**b**,**c**) Relative mRNA expression of *TNF-α* and *IL-6* at indicated time points in placenta or embryo, respectively (n = 3 pregnant rats in group). Indications of Con and LPS are the same as described in Fig. 2. Error bars represent S.D. *p < 0.05 and **p < 0.01, LPS versus Con at the same time point, respectively.

To further resolve whether the early life NF-κB activation in fetal thoracic aorta was triggered by the direct damage from LPS or by the indirect damage caused by maternal derived pro-inflammatory cytokines exposure, we assessed the protein or mRNA levels of tumor-necrosis factor-α (TNF-α) and interleukin-6 (IL-6) in maternal serum, placenta, as well as the embryo at different time points after the last i.p. injection of LPS. We found that the expression levels of TNF-α and IL-6 in maternal serum and placenta were immediately increased 2 hours (2 h) after last time LPS administration (Fig. 4a,b). However, only *IL-6* mRNA expression in the fetus was significantly increased after 48 h of LPS administration (Fig. 4c). The delayed *IL-6* mRNA elevation in fetus, in comparison to its rapid and robust increases in maternal serum and placenta, suggests that the damage on embryonic development might be caused indirectly by maternal derived pro-inflammatory cytokines exposure, rather than directly from *in utero* LPS.

Collectively, our data demonstrate that both PI3K-Akt-mediated degradation of IκBα and impaired NF-κB self-negative feedback loop on newly IκBα re-synthesis implicates in IκBα down-regulation and NF-κB over-activation in conduit arteries, leading to hypertension in the offspring exposed to an inflammatory stimulus during the prenatal period.

### Prenatal or postnatal administration of the NF-κB inhibitor, PDTC, prevents elevated blood pressure in inflammation-induced PPH rats

**Figure 5:**
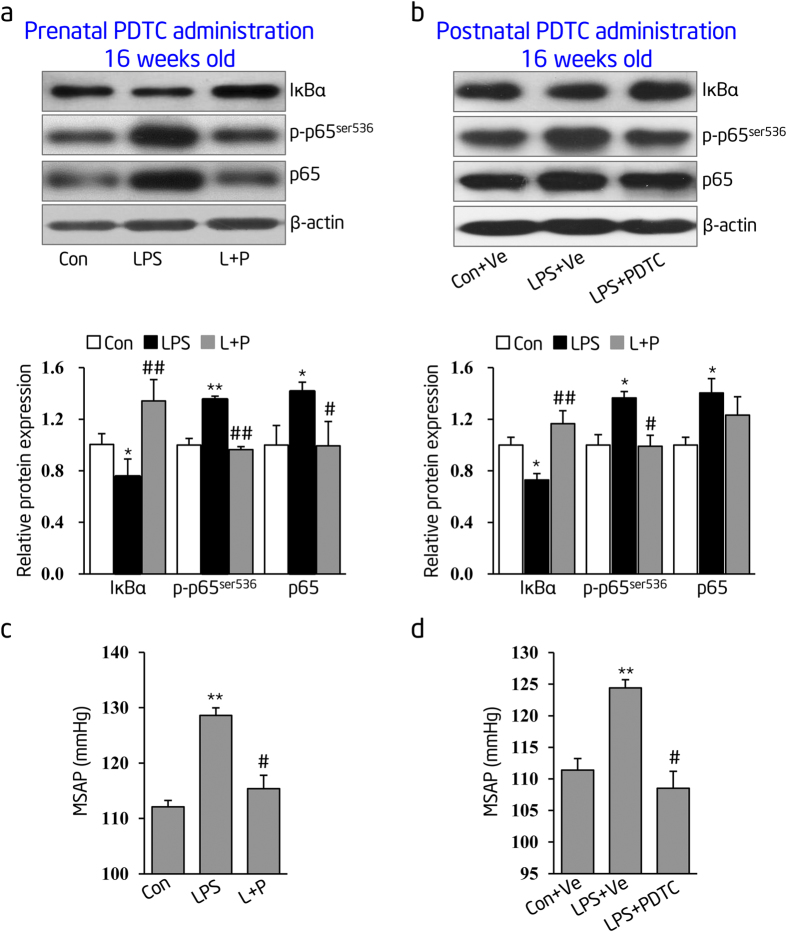
Prenatal or postnatal administration of specific IκBα degradation inhibitor, pyrollidine dithiocarbamate (PDTC), reverses blood pressure elevation in prenatal inflammation-induced PPH rats. (**a**,**b**) IκBα, phosphorylated (p)-p65 and total p65 protein expression were assessed by immunoblotting in thoracic aortas of 16-week-old offspring from prenatal LPS stimulation simultaneously with prenatal PDTC administration (**a**) or postnatal PDTC daily administration from 7 to 16 weeks old (**b**), respectively. Representative blot (top panel) and statistical data of relative densitometry (bottom panel), normalized by β-actin, were shown (n = 6 offspring in each group). (**c**,**d**) Mean systolic artery pressure (MSAP) was measured by standard tail-cuff method in 16-week-old conscious offspring from prenatal PDTC administration simultaneously with LPS stimulation (**c**) or post-birth PDTC daily administration from 7 to 16 weeks old (**d**) (n = 8 male and 8 female offspring in each group). Con indicates prenatal vehicle control; LPS, prenatal LPS stimulation; L + P, prenatal LPS plus PDTC administration; Con + Ve, offspring from Con group accepted saline daily from 7 to 16 weeks old as vehicle control; LPS + Ve, offspring from LPS group accepted saline daily from 7 to 16 weeks old as model control; LPS + PDTC, offspring from LPS group accepted PDTC daily from 7 to 16 weeks old. Error bar represents S.D. *p < 0.05 or **p < 0.01, LPS versus Con or LPS + Ve versus Con + Ve, res*p*ectively; ^#^*p* < 0.05 or ^##^*p* < 0.01, L + P versus LPS or LPS + PDTC versus LPS + Ve, respectively.

To further explore the physiological role of NF-κB activation on the development of hypertension in offspring of prenatal inflammatory stimulation, we used the inhibitor of IκBα degradation, PDTC, *in vivo* to block NF-κB activation simultaneously with LPS stimulation or postnatally in adulthood. Treatment with PDTC started at postnatal week 7 and evaluated the NF-κ B activity and blood pressure at postnatal week 16. Our results identified a decrease in IκBα, with reported increases in phosphorylated p65 (p-p65) and total p65 protein levels in thoracic aortas of prenatal LPS-stimulated offspring at the age of 16 weeks (Fig. 5a**, LPS group** and Fig. 5b**, LPS + Ve group**). Conversely, elevated IκBα, decreased p-p65 and total p65 protein expression in thoracic aortas after PDTC administration at both prenatal (Fig. 5a**, L + P group**) and postnatal stage (Fig. 5b**, LPS + PDTC group**). Our data suggests that NF-κB activation in the conduit artery of prenatal LPS-stimulated offspring is a lifelong persistent event. In addition, our data also suggests that PDTC efficiently inactivates NF-κB signaling in the offspring’s thoracic aortas. Our published research in this area had shown that prenatal LPS stimulation leads to development of offspring’s hypertension with no gender differences^15^. In the current study, we were interested in exploring whether persistent NF-κB activation was associated with the development of hypertension in both male and female offspring. We found that prenatal PDTC administration significantly abolished offspring’s mean systolic artery pressure (MSAP) elevation induced by prenatal LPS stimulation (**L + P group**), compared with that in offspring of prenatal LPS stimulation alone (**LPS group**) (LPS, 129 ± 1 mmHg versus L + P, 115 ± 2 mmHg; 8 male and 8 female offspring for each group, *p* < 0.05) (Fig. 5c). Our results also demonstrated at postnatal 16 weeks old, daily PDTC administration inhibited offspring’s MSAP elevation induced by prenatal LPS exposure (LPS + Ve, 124 ± 1 mmHg versus LPS + PDTC, 109 ± 3 mmHg; 8 male and 8 female offspring for each group, *p* < 0.05) (Fig. 5d). However, postnatal PDTC treatment had no effect on MSAP in control offspring (data not shown). These results indicate that early life and persistent NF-κB activation is highly associated with the offspring’s gradual elevation in blood pressure.

### Prenatal PDTC administration alleviates RAS over-activity and improves endothelial dysfunction in thoracic aortas of inflammation-induced PPH rats

**Figure 6:**
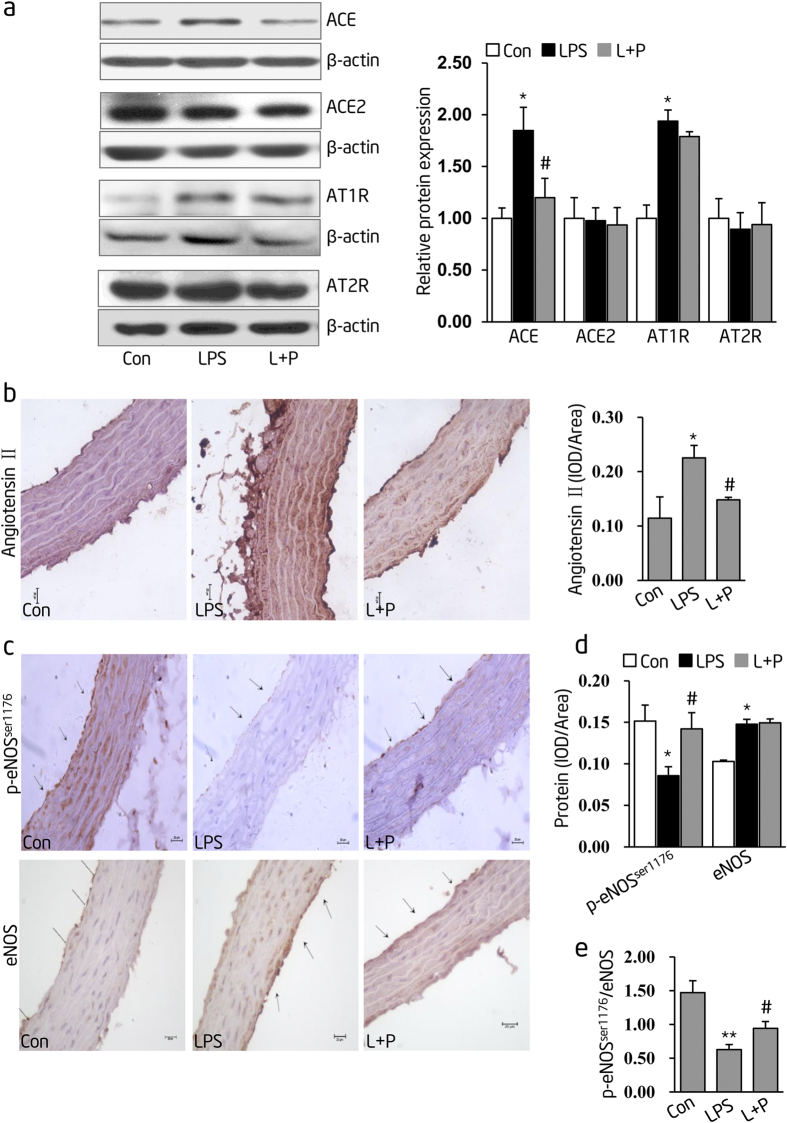
Prenatal PDTC administration prevents renin-angiotensin system (RAS) over-activity and endothelial dysfunction in thoracic aortas of inflammation-induced PPH rats at the age of 16 weeks. (**a**) Angiotensin I-converting enzyme (ACE), ACE2, angiotensin II type-1 receptor (AT1R) and angiotensin II type-2 receptor (AT2R) protein levels were determined by immunoblotting. Representative plots of 1 from 6 offspring in each group (left panel) and statistical data of relative densitometry (right panel), normalized by β-actin, were shown. (**b**) Angiotensin II protein level was determined by immunohistochemistry staining. Representative pictures pooled from each group were shown (n = 4 offspring in each group) (left panel) and interoptical density (IOD) value was quantified by using Image-Pro Plus software (right panel). (**c**) The protein expressions of phosphorylated endothelial nitric oxide synthase (p-eNOS^ser1176^) (upper panel) and total endothelial nitric oxide synthase (eNOS) (lower panel) in thoracic aortas were determined by immunohistochemistry staining from 16-week-old offspring. Vessel wall, nearby the arrow direction, represents endothelium. Representative pictures, selected from 1 out of 4 offspring in each group, were shown. (**d**) IOD value of p-eNOS^ser1176^ and eNOS from (**c**) were quantified by using Image-Pro Plus software and shown as mean ± S.D. (**e**) Summarized data of the ratio of p-eNOS^ser1176^ to eNOS IOD in each group, calculated from (**d**), were shown as mean ± S.D. Indications of Con, LPS and L + P are as described in Fig. 4. Error bars represent S.D. *p < 0.05 and **p < 0.01, LPS versus Control; ^#^*p* < 0.05, L + P versus LPS.

To explore the mechanisms by which PDTC inhibited the offspring’s blood pressure elevation, we first determined the local RAS activity in thoracic aortas since it is known to contribute to the chronic process of secondary structural damages during hypertension development^25^. The protein levels of RAS family members, such as Ang II, ACE, ACE2, AT1R and AT2R, were determined by immunostaining or immunoblotting. We found that prenatal LPS stimulation increased protein levels of ACE, AT1R and Ang II, but had no effect on ACE2 and AT2R in thoracic aortas (Fig. 6a,b**, LPS group**). The increase in ACE and Ang II protein levels by prenatal LPS exposure was obviously abolished by prenatal PDTC treatment (Fig. 6a,b, **L + P group**). However, prenatal PDTC treatment did not significantly influence the protein expression levels of ACE2, AT1R and AT2R (Fig. 6a**, L + P group**).

Furthermore, we also assessed the protein levels of p-eNOS^ser1176^ and total eNOS, known markers of vascular endothelium function^37^, by immunostaining in thoracic aortas. The p-eNOS^ser1176^ level was significantly decreased in thoracic aortas of prenatal LPS-stimulated offspring (Fig. 6c,d, **LPS group**); however, this decrease in p-eNOS^ser1176^ expression was also abolished by prenatal PDTC administration (Fig. 6c,d, **L + P group**). Although the eNOS protein level was unchanged in both L + P group and LPS group (Fig. 6c,d), the ratio of p-eNOS^ser1176^ to total eNOS expression was decreased in thoracic aortas of prenatal LPS-stimulated offspring (LPS group) whereas it was increased by prenatal PDTC administration (L + P group) (Fig. 6e).

### Postnatal long-term PDTC administration prevents offspring from RAS over-activity and endothelial dysfunction at thoracic aortas of inflammation-induced PPH rats

**Figure 7:**
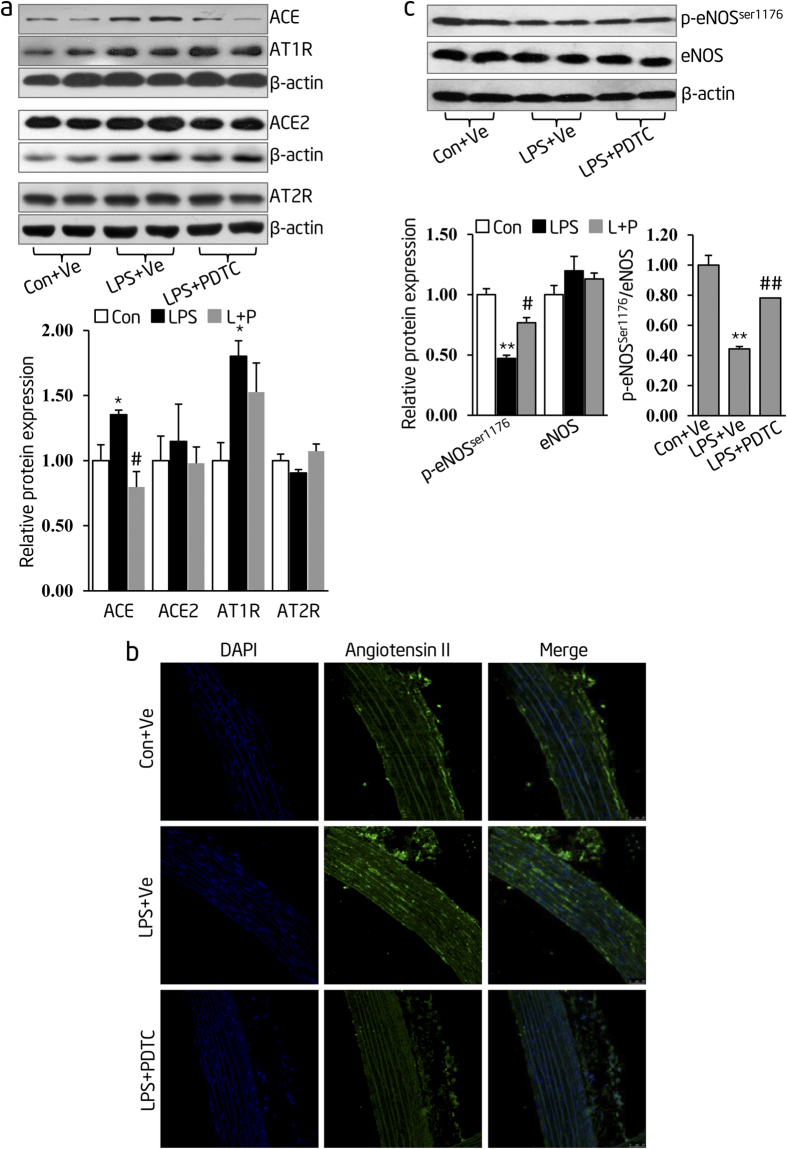
Postnatal persistent PDTC administration alleviates RAS overactivation and endothelial dysfunction in inflammation-induced PPH rats. (**a**) After 9 weeks of PDTC administration started at postnatal week 7, protein abundance of ACE, ACE2, AT1R and AT2R were determined by immunoblotting in thoracic aortas of 16-week-old offspring. β-actin was taken as the internal control. Representative plots of 2 from 6 offspring in each group were shown (upper panel). Statistical data of relative densitometry, normalized by β-actin, were shown as mean ± S.D (lower panel). (**b**) Offspring were treated as described in (**a**) and Angiotensin II protein level was determined by immunofluorescence staining. Representative pictures selected from each group were shown (n = 4 offspring in each group). (**c**) Offspring were treated as described in (**a**), and p-eNOS^ser1176^ and total eNOS expression were determined by immunoblotting. Representative plots of 2 from 6 offspring in each group were shown (upper panel). Summarized data of p-eNOS, eNOS relative densitometry and the ratio of p-eNOS to eNOS densitometry in each group were shown as mean ± S.D (lower panel). Indications of Con + Ve, LPS + Ve and LPS + PDTC are as described in Fig. 4. Data were shown as mean ± S.D. Error bars represent S.D. *p < 0.05 and **p < 0.01, LPS + Ve versus Con + Ve; ^#^*p* < 0.05 and ^##^*p* < 0.01, LPS + PDTC versus LPS + Ve.

In this segment of our research, we wanted to determine the protective effect of PDTC on offspring’s thoracic aortas in the model of postnatal PDTC administration. We assessed the offspring’s RAS activity by measuring the expression levels of relevant proteins (Ang II, ACE, ACE2, AT1R and AT2R) in thoracic aortas of offspring at the age of 16 weeks after 9 weeks of daily PDTC administration that started at postnatal week 7. The protein levels of ACE and AT1R were increased in thoracic aortas of prenatal LPS-stimulated offspring (**LPS + Ve group**) compared to those in control (**Con + Ve group**). Postnatal PDTC treatment abolished the changes of ACE but not AT1R protein expression in thoracic aortas of offspring with prenatal LPS exposure (**LPS + PDTC group**). There was no significant difference of ACE2 and AT2R expression among these groups (Fig. 7a). We found higher Ang II expression in thoracic aortas of prenatal LPS-stimulated offspring (**LPS + Ve group**) compared to controls (**Con + Ve group**). Interestingly, this elevated expression of Ang II was attenuated by postnatal long-term PDTC administration (**LPS + PDTC group**) (Fig. 7b). On the other hand, the expression of p-eNOS^ser1176^ protein was decreased in thoracic aortas of prenatal LPS-stimulated offspring (**LPS + Ve group**) compared with that of controls (**Con + Ve group**), suggesting that prenatal LPS exposure led to the suppressed level of p-eNOS^ser1176^ protein at an older age. Interestingly, this decrease in p-eNOS^ser1176^ protein expression mediated by prenatal LPS exposure was efficiently rescued by the PDTC treatment (**LPS + PDTC group**). The ratio of p-eNOS ^ser1176^ to total eNOS showed a similar trend among these groups since there was no change in the expression of total eNOS protein under these conditions (Fig. 7c).

Our previous report have demonstrated that prenatal PDTC treated control offspring only showed transient increased Ang II expression in kidney 1 day after birth, but soon reversed to the same level in adult^16^ and the postnatal PDTC treatment did not have any obvious effects on RAS activity in control offspring (data not shown). Thus, our data supports a newly defined network that early life NF-κB activation is responsible for RAS over-activity and endothelium dysfunction in conduit artery of PPH rats.

### Prenatal LPS stimulation shows no effect on RAS mRNA transcript in thoracic aortas of inflammation-induced PPH rats at early life

**Figure 8:**
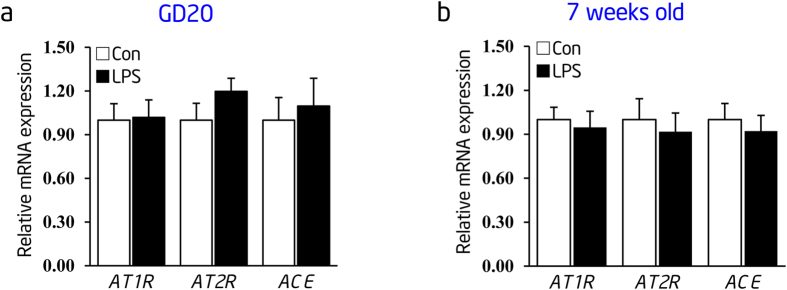
Expression of RAS components mRNA remains unchanged in thoracic aortas of inflammation-induced PPH rats at early life stage. (**a**,**b**) Expression of *AT1R*, *AT2R, ACE* and *β-actin* at the mRNA level in thoracic aortas was determined by real-time RT-PCR at gestational day 20 and postnatal week 7. *β-actin* was taken as internal control. Indications of Con and LPS are as described in Fig. 2. Error bars represent S.D. (n = 6 offspring in each group).

To further determine the expression changes of RAS components in offspring’s thoracic aortas after prenatal LPS stimulation, we measured the transcript levels of AT1R, AT2R, and ACE in offspring’s thoracic aortas at GD 20 or postnatal week 7 by real-time RT-PCR, respectively. Interestingly, there was no significant change in the mRNA expression of RAS components in thoracic aortas of prenatal LPS-stimulated offspring at both GD 20 and postnatal week 7 (Fig. 8a,b). The upregulation in RAS activity was delayed in comparison to NF-κB activation in thoracic aortas of prenatal LPS-stimulated offspring. This suggested that early life and persistent NF-κB activation is a critical factor for the elevation in RAS activity in the local vasculature of inflammation-induced PPH rats, although other risk factors may also exist.

### Prenatal LPS exposure leads to resistance artery lesion but not NF-κB activation nor RAS over-activity in inflammation-induced PPH rats at the postnatal age of 16 weeks

**Figure 9:**
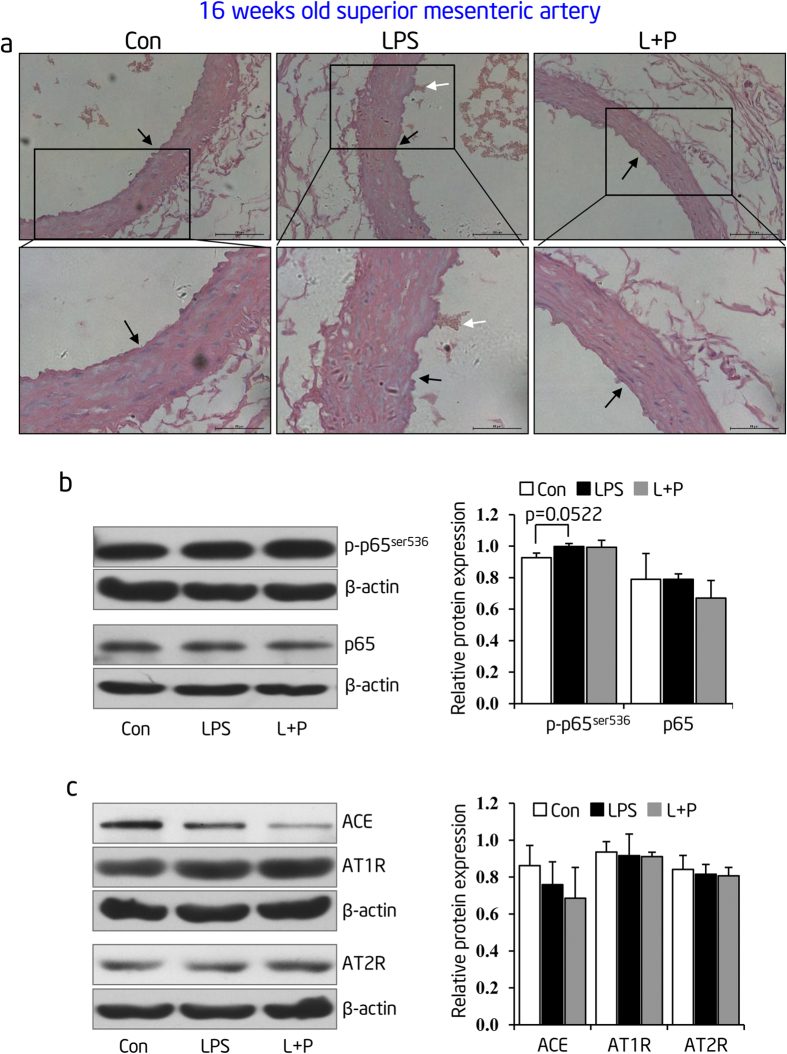
Prenatal LPS stimulation leads to structural damages of superior mesenteric arteries without NF-κB activation and RAS over-activity. (**a**) Hematoxylin-eosin (H&E) staining of superior mesenteric arteries of 16-week-old offspring. Vessel wall, nearby the black arrow direction, represents endothelium. White arrows indicate the detached endothelium (n = 4 in each group). (**b**,**c**) Protein levels of phosphorylated (p)-p65, total p65 (**b**) and RAS family (**c**) were determined by immunoblotting in superior mesenteric arteries of 16-week-old offspring, respectively. Representative plots of 1 from 6 offspring in each group were shown (left panel). Statistical data of relative densitometry, normalized by β-actin, was shown as mean ± S.D (right panel). Error bars represent S.D.

Because damage of resistance artery structure plays a critical role in the progression of EH, we focused our efforts on detecting the pathological changes of superior mesenteric arteries in prenatal LPS-stimulated offspring at the age of 16 weeks. As expected, we observed obvious enlargement of the media muscular layer existed in superior mesenteric arteries of prenatal LPS-stimulated offspring (**LPS group**) at 16 weeks of age. In addition, we also detected that some endothelial layers were detached or even desquamated from the basement membrane in superior mesenteric arteries of prenatal LPS-stimulated offspring (**LPS group**). In contrast, prenatal PDTC administration prevented the enlargement of the media muscular layer and lesion of endothelial layer in superior mesenteric arteries that were caused by prenatal LPS exposure (Fig. 9a).

We previously reported inconsistent changes of ACE expression in conduit arteries and resistance arteries in spontaneous hypertensive rats^26^. As such, it suggests a tissue specific role of RAS activity during the development of hypertension. Henceforth, this prompted us to determine NF-κB and RAS activity in resistance arteries after prenatal LPS exposure by detecting the protein expression of p-p65, p65, and the targeted RAS family of proteins. We did not observe any significant change in any of these targeted proteins (Fig. 9b,c). The consistent changes between NF-κB and RAS activity in conduit and resistance arteries, taken together with the finding that RAS activity upregulation was delayed in comparison to NF-κB activation in conduit arteries (Fig. 8), suggest that NF-κB upregulating RAS activity in conduit artery is critical to aortic dysfunction and hypertension development.

## Discussion

The present study demonstrated that prenatal inflammatory exposure caused conduit but not resistance artery IκBα/IκBβ imbalance, leading to early life NF-κB activation. This dyshomeostasis evoked progressive dysfunction of RAS in adult offspring’s conduit artery, and subsequently aggravated blood pressure resulting in the development of hypertension. The major findings of this study is that prenatal LPS exposure caused a NF-κB activation, characterized by decreased IκBα level through both PI3K-Akt-mediated degradation of IκBα and less newly synthesized *IκBα* mRNA by impaired NF-κB self-negative feedback loop, from fetus to adult. As such, this indirect damage on the fetus is mainly caused by the fetal exposure of maternal-derived pro-inflammatory cytokines. In addition, we have also found that prenatal or postnatal application of specific IκBα degradation inhibitor PDTC prevented the offspring’s blood pressure elevation and endothelium dysfunction. Furthermore, we showed that prenatal LPS exposure led to higher levels of ACE and Ang II in aortic tissue, which could be abolished by both prenatal and postnatal PDTC application. Finally, we found that prenatal LPS exposure resulted in resistance artery lesion but not NF-κB activation or RAS over-activity in offspring at the age of 16 weeks. These findings support our hypothesis that early life NF-κB dyshomeostasis in conduit artery, induced by prenatal inflammatory stimulation, is predictive of EH development in adult.

Inflammation plays an important role in vascular dysfunction and hypertension^38^. Recent research has focused on the effect of these inflammatory mediators in specific regard to their effects on the conduit artery; however, their effects during the progression of hypertension are largely unknown. The current study focuses on the effects of prenatal inflammatory stimuli on the development of hypertension. We identified that prenatal exposure to inflammatory stimuli causes higher levels of pro-inflammatory factors and NF-κB activation in the fetus (48 h after the last LPS administration) as well as in thoracic aortas of 7-week-old offspring. We also found that NF-κB p65 DNA binding activity and expression of pro-inflammatory factor TNF-α were significantly higher in the thoracic aorta of prenatal inflammation stimulated offspring at the age of 7 weeks. As persistent NF-κB activation is a nexus for inflammation response and tissue damage^39^, a deep understanding on how distinct pathways activate or inhibit NF-κB activity may be indispensable for us to search and identify the potential targets for treatment or prevention of certain human diseases. NF-κB activation is reported to exist in kidney^40^,^41^ and heart^42^ of older spontaneous hypertensive rats with unknown mechanisms. In the present study, using a classic immune-inflammation stimulator LPS during the second trimester of fetal development, we found that persistent NF-κB activation can be programed during the fetal development by inflammatory challenge, and blocking NF-κB activation by PDTC could prevent hypertension development in offspring of prenatal inflammatory stimulation. Thus, we believe that early life and persistent NF-κB activation programed by prenatal inflammatory stimuli is a critical factor for inflammation ignition and maintenance in the early stage of hypertension development.

NF-κB and IκB proteins play as an integrated network in regulating NF-κB activation. At rest state, IκBα maintains NF-κB in an inactive form in the cytoplasm by blocking the nuclear localization signals of NF-κB proteins, while IκBβ can sequester p65 in the cytoplasm by masking NF-κB nuclear localization sequences^43^,^44^. Upon stimulation, IKKs phosphorylate the classical IκB proteins IκBα, IκBβ and IκBε at specific serine residues, which lead to their proteasomal degradation, and nuclear translocation of free NF-κB. However, NF-κB-dependent transcription of IκBα gene leads to rapid re-synthesis of the IκBα protein, and inhibition of NF-κB-dependent transcription at nucleus^45^. At the same time, newly synthesized IκBβ shows a hypo-phosphorylated form, which mainly stays in the nucleus, binds DNA with p65 and c-Rel. This DNA-bound NF-κB/IκBβ complexes are resistant to IκBα, and can prolong pro-inflammatory gene expression^46^,^47^. In the current study, we found that both mRNA and protein levels of IκBα were diminished in aortic tissue from fetus to adult after prenatal inflammatory exposure, whereas no significant changes existed in IκBβ expression. Mechanically, we found no expression changes of IKKs, but increased PI3K-Akt activation mediated degradation of IκBα protein and impaired NF-κB self-negative feedback loop on newly IκBα synthesis implicated in IκBα down-regulation in conduit arteries of prenatal inflammation-stimulated offspring. This indicates a specific gene re-imprinting of IκBα after prenatal inflammatory stimulation is responsible for impaired NF-κB clearance capacity and activation.

The RAS is described as a cascade of biochemical reactions, whose activity is essential for cardiovascular homeostasis^48^. The increased Ang II level leads to the expression of IL-6, MCP-1 and TNF-α in monocytes in vascular tissue, which contributes to the vascular inflammation leading to vascular damage^49^. Our previous studies demonstrated that systemic levels of Ang II^16^, NO and endothelin 1^15^ remained unchanged but the levels of renal renin, Ang II expression^16^ and thoracic aortas’ Ang II, AT1R^30^ were significantly increased in prenatal inflammation-induced PPH rats. The present study found that ACE over-expression was responsible for increased Ang II expression in thoracic aortas of prenatal inflammation stimulated offspring. Interestingly, increased Ang II, AT1R, ACE expression and NF-κB activation didn’t exist in the resistance artery of prenatal LPS-stimulated offspring, which is consistent with previous finding in spontaneous hypertensive rats^26^. This similarity with spontaneous hypertensive rats further supported our idea that prenatal inflammatory stimulation programed hypertension could also be good model to study EH in the future.

Increased tissue Ang II favors the expression of regulatory, structural, and cytokine genes through the activation of NF-κB signal pathway in EH, which plays important roles in long-term control of blood pressure, vascular remodeling, cardiac hypertrophy and inflammation^50^,^51^. Our current study found that NF-κB activation, occurring in thoracic aortic tissue in both fetus and adult, took place prior to the expression changes of RAS components. Increased ACE and Ang II expression were reversed by both prenatal and postnatal PDTC administration. However, we could not find any direct evidence of increased NF-κB binding to ACE promoter in thoracic aortas of offspring from prenatal exposure to LPS (data not show). Our study demonstrates that this delayed RAS over-activity is indirectly caused by NF-κB activation through the pro-inflammatory nexus preceding hypertension development in PPH rats. Intriguingly, prenatal or postnatal PDTC application predominantly reversed ACE but showed no effect on AT1R receptor expression. This might be the reason that PDTC blocked the reported positive feedback loop between NF-κB and RAS (NF-κB → ACE → Ang II → NF-κB)^52^. Together with the finding that the consistent changes between NF-κB and RAS activity only in conduit but not in resistance arteries, we demonstrate that prenatal PDTC treatment can block the initial factor of NF-κB activation. We also demonstrate that postnatal PDTC treatment can break the RAS positive feedback, both of which could prevent the development of prenatal programed hypertension.

Endothelial cells release endothelial-relaxing factors and therefore have a critical role in vasodilation, inhibition of platelet aggregation and monocyte invasion. Endothelium dysfunction is associated with a variety of diseases including atherosclerosis, diabetes mellitus, coronary artery disease, hypertension and hypercholesterolemia^53^. In the current study, we found decreased eNOS phosphorylation level in conduit artery of PPH rats at 16 weeks old, which was restored by PDTC administration at fetus or after birth. The activation of NF-κB → ACE → Ang II → Reactive oxygen species signal pathway plays a major role in the pathogenesis of endothelium dysfunction^54^. We previously found no significant vasodilation reactivity at the age of 12 weeks, which indicated the proper endothelium function at that time point^18^. This suggests that the endothelium dysfunction might be a secondary event of NF-κB → ACE → Ang II → Reactive oxygen species → NF-κB positive feedback signal pathway during the development of hypertension in prenatal inflammation-induced offspring.

In summary, the present study has established a new and direct link between programmed NF-κB dyshomeostasis, early life NF-κB activation and RAS over-activity in conduit artery in prenatal inflammation-induced PPH rats. Following prenatal inflammatory stimulation, early life and adult lifelong NF-κB activation ignites the NF-κB → ACE → Ang II → NF-κB positive feedback loop in conduit artery, which in turn predisposes to aortic dysfunction and development of prenatal inflammation programmed hypertension. Since prenatal inflammatory exposure is an important unresolved public health problem, the present findings provide a new notion to reduce the incidence of adult hypertension at an early life stage. As NF-κB dyshomeostasis might be predictive of PPH, it would be beneficial to identify safe agents to efficiently modulate the NF-κB signaling pathway for prevention or treatment of EH starting at the neonatal stage.

## Materials and Methods

### Materials

LPS (from Escherichia coli 026:B6, Cat log: L8274), PDTC (Cat log: P8765), and other chemicals were obtained from Sigma-Aldrich.

### Animals

Nulliparous time-dated pregnant SD rats were purchased from the Animal Centre of Third Military Medical University (Chongqing, China). All animals took standard laboratory rat chow and tap water ad artitrium. Rats were housed individually in a room at constant temperature (24 °C) and under a 12-h light–dark cycle until parturition. Pups were raised with a lactating mother until 4 weeks of age, at which time they were weaned to cages containing four pups for each^15^. This study was conducted in accordance with the principles outlined in the National Institutes of Health Guide for the Care and Use of Laboratory Animals. All procedures and protocols were approved by the local animal ethics committee at Third Military Medical University.

#### Study I: Prenatal PDTC study

Time-dated pregnant (GD 8) SD rats (250 g to 300 g) were randomly divided into three groups (n = 8 in each group) as described previously^16^. The pregnant rats in these groups were intraperitoneally (i.p) administered with saline (**Control group**), LPS 0.79 mg/kg **(LPS group)**, or LPS 0.79 mg/kg plus PDTC 0.609 mmol/kg **(L + P group)**, respectively. LPS was given on GD 8, 10 and 12, whereas PDTC was given daily from GD 8 to 14. The pregnant rats in the LPS group were given saline injection on GD 9, 11, 13 and 14, and pregnant rats in the control group were given saline daily from GD 8 to14. After delivery, the size of the litter was reduced to 8 for each mother by random selection to avoid the effect of nutritional disproportion.

#### Study II: Postnatal PDTC study

For postnatal PDTC administration, pups from the aforementioned LPS group in Study I were randomly separated into two groups and daily i.p administered with PDTC (0.609 mmol/kg, **LPS + PDTC group**) or with vehicle saline (**LPS + Ve group**) from postnatal week 7 to week 16. Aforementioned control group offspring in Study I received saline daily starting at postnatal week 7 were taken as vehicle control (**Con + Ve**).

### Collection of serum and tissues

Blood samples from pregnant rats were obtained *via* heart puncture 2 h after the last LPS i.p injection. After clotting for a half hour and then centrifuging at 3000 rpm for 20 min, the serum was collected and stored at −80 °C for later use.

Placental and embryonic tissues were collected and then stored at RNA later solution (Tiangen Biotech, Beijing, China) at −80 °C from pregnant rats that were anaesthetized at indicated time points after the last LPS i.p injection.

### Real-time RT PCR

Real-time RT-PCR was performed as previously described^16^. Briefly, the thoracic aortic *p65*, *IκBα*, *IκBβ*, *IKKα*, *IKKβ*, *IKKγ, TNF-α* and *IL-6* mRNA expressions were assessed by real-time RT-PCR when the offspring were at gestational day 20 or postnatal week 7, respectively. Total RNA was extracted from thoracic aortas using Trizol (Roche Molecular Biochemicals, Mannheim, Germany) and total RNA (1 μg) was then reverse-transcribed into cDNA using a First Stand cDNA Synthesis Kit (Toyobo, Osaka, Japan). Primer sequences for real-time RT-PCR were listed in Table 1. Each real-time PCR reaction was carried out in a total volume of 20 μl with Quanti Tect SYBR Green PCR Master Mix (MJ Research, Waltham, MA, USA) according to the following conditions: 2 min at 95 °C, 40 cycles at 95 °C for 10 s, 60 °C for 15 s, 72 °C for 20 s, using ABI Prism 7700 sequence detection system (Applied Biosystems, Foster City, CA, USA).

Relative mRNA expression was calculated by normalizing the relative gene expression to the control group after normalized the cycle threshold value by the internal control *β-actin*.

### Immunoblotting

Protein expression in thoracic aortas and superior mesenteric arteries was determined by immunoblotting, as described previously^16^. Briefly, thoracic aortas and superior mesenteric arteries were homogenized in a T-PER tissue protein extraction reagent (Thermo-Pierce, Rockford, IL, USA) with protease inhibitor cocktails (Sigma-Aldrich, St. Louis, MO, USA). After quantitative determination of protein concentration in each sample by BCA Protein Assay Kit (Bio-Rad, Hercules, CA, USA), 20 μg proteins of each sample were separated by 4–15% SDS-PAGE and transferred to a nitrocellulose membrane. After blocked by 5% nonfat dry milk for 1 hour at room temperature, the membranes were then incubated with anti-IκBα (L35A5), anti-IκBβ (Polyclonal), anti-phosphorylated (p)-p65^ser536^ (93H1), anti-p65 (D14E12), anti-p-Akt^ser473^, anti-p-GSK-3β^ser9^ (D85E12), anti-Akt (C73H10), anti-GSK-3β (27C10) (Cell signaling Technology, Beverly, MA, USA); anti-ACE (N-20), anti-ACE2 (Polyclonal) (Santa Cruz Biotechnology, Santa Cruz, CA, USA); anti-AT1R (Ployclonal), anti-AT2R (EPR3876), anti-p-eNOS^ser1176^ (Polyclonal), anti-eNOS (Polyclonal) (Abcam, Cambridge, UK), or anti-β-actin (AC-15) (Sigma-Aldrich) antibodies overnight at 4 °C, followed by a secondary antibody incubation. Proteins were visualized with enhanced chemiluminescence reagents, and the blots were exposed to hyper film. The single band for β-actin, under multi-antigens, is a representative picture of several different blots with the same samples. Results were quantified by using Quantity-one software (Bio-Rad).

### Detection of NF-κB p65 DNA binding activity

Nuclear proteins of thoracic aorta from 7-week-old offspring were isolated by using a nuclear and cytoplasmic protein extraction kit (Beyotime, Haimen, China) according to the manufacturer’s instruction. Protein concentration was determined by BCA Protein Assay Kit (Bio-Rad) and 50 μg nuclear proteins were used to measure the NF-κB p65 DNA binding activity using a commercial NF-κB EZ-TFA transcription factor assay colorimetric kit (Merck Millipore, Billerica, MA, USA) according to the manufacturer’s protocol.

### Quantification of TNF-α and IL-6 protein in serum and thoracic aorta

TNF-α and IL-6 protein levels in serum were assessed by radioimmunoassay (Beijing North Institute of Biological Technology, Beijing, China) according to the manufacturer’s instruction.

Thoracic aortas were homogenized to detect the expression levels of TNF-α and IL-6 by ELISA (R&D Systems, Abington, UK) according to the manufacturer’s protocol.

### ChIP assay

ChIP assay was performed with a ChIP assay kit (Upstate Biotechnology, Lake Placid, NY), according to the manufacturer’s instructions, as described previously^55^,^56^. In brief, 30 mg thoracic aortic tissues for each IP were cut into small pieces (1–2 mm^2^) and fixed for 10 minutes at RT with 37% formaldehyde, followed by quenching with 10× glycine. Fixed tissue pieces were disaggregated with Dounce homogenizer, then pelleted, washed with PBS, lysed and sonicated with ultrasonic crusher (fifteen cycles of 15-s pulses at 90% max amplitude, Diagenode). An equal amount (5 μg) of rabbit monoclonal anti-p65 Abs or normal rabbit IgG Abs (Cell Signaling Technology) were used to precipitate the cross-linked DNA/protein complexes. DNA precipitated by the anti-p65 or the normal rabbit IgG Abs was quantified by real-time PCR, and values were normalized to input DNA. The primers used in the analysis of rat *IκBα* (*NFKBIA*) promoter are as follows: −275 to −266 bp: (F)-5′-CCGTCGTCGCAGAAATTTAG-3′; (R)-5′-GTTCTACAAGAGCCCTTCTTT-3′. −181 to −172 bp: (F)-5′- GGGAAAGAAGGGCTCTTGTA-3′; (R)-5′-CCTCGACTGAGAAGCCTAAA-3′; −31 to −21 bp: (F)-5′- AAAGTTCCCTGTACATGACC-3′; (R)-5′-GGAATTTCCAAGCCAGTCAG-3′.

### Blood pressure measurement

MSAP was measured in 16 conscious offspring (8 male and 8 female randomly chosen from 8 different lactating mothers) from each group at the age of 16 weeks using the standard tail-cuff method (ML125; Powerlab, AD Instruments), as described previously^15^. Rat was placed inside a warming chamber (~34 °C) for 15 min before taking artery blood pressure measurement and was then placed in plastic restraints. A cuff with a pneumatic pulse sensor was attached to the tail. All rats were allowed to habituate to this procedure 7 days before the experiments. The rats were trained at least three times before formal measurement. In each rat, MSAP was calculated from three consecutive recordings of artery systolic blood pressure. The investigators were blinded for measuring the blood pressure.

### Immunohistochemistry and immunofluorescence

Thoracic aortas Ang II, eNOS and phosphorylated-eNOS expression were identified by immunohistochemical (IHC) staining or immunofluorescence staining as described previously^16^,^18^. Histological structure of superior mesenteric arteries was determined by standard hematoxylin-eosin (H&E) staining. Arteries were incubated in 4% paraformaldehyde for 1 week and then embedded in paraffin wax. The sections (4 μm) were rinsed and rehydrated in PBS for 5 min. An indirect immunoperoxidase staining technique was performed using the two-step IHC detection reagent according to the manufacturer’s protocol (Beijing Zhongshan Golden Bridge Biotechnology). Primary antibodies are as follows: anti-Ang II (Ployclonal) (Novus, A-8 Litterton, CO, USA), anti-eNOS (3/eNOS/NOS Type III) (BD Transduction Laboratories, Bedford, MA, USA) and anti-p-eNOS^ser1176^ (Polyclonal) (Abcam). Peroxidase activity was visualized using a DAB kit. Slices were viewed with an Olympus BH-2 microscope and images were captured with an attached SPOT digital camera imaging system. The expression levels of Ang II, eNOS and p-eNOS were quantified by interoptical density value (IDO) by using Image-Pro Plus software (Media Cybernetics, Rockville, MD, USA).

For Ang II immunofluorescence, goat anti-rabbit IgG H&L (FITC) antibodies (Abcam) were used for an additional 60 minute incubation after the primary antibody incubation and washing. Sections were then incubated with 1 mg/ml DAPI for 30 min, washed three times with PBS, and mounted into Vectashield^®^ mounting medium (Vector Laboratories, Burlingame, CA). The coverslips were visualized under a Leica confocal laser scanning microscope (Leica).

The histological structure of superior mesenteric artery was analyzed by standard H&E staining after sections were dewaxed and rehydrated.

The investigators were blinded for acquiring the images.

### Statistical analyses

For unpaired comparisons of two independent groups, unpaired stutent’s t-test was used. One-way ANOVA model was used for multiple comparisons. Data are expressed as mean ± S.D. *p* values were adjusted for multiple comparisons using Bonferroni method. All tests are two-sided. A *p* < 0.05 was considered significant.

## Additional Information

**How to cite this article**: Deng, Y. *et al.* Prenatal inflammation-induced NF-κB dyshomeostasis contributes to renin-angiotensin system over-activity resulting in prenatally programmed hypertension in offspring. *Sci. Rep.* **6**, 21692; doi: 10.1038/srep21692 (2016).

## Figures and Tables

**Figure 1:**
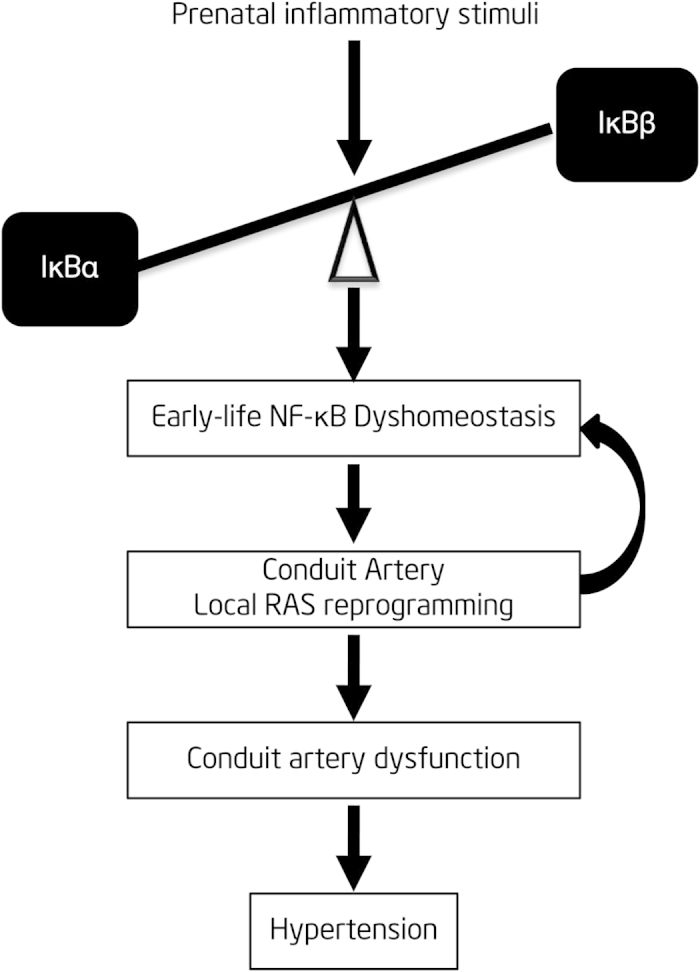
Hypothesis of the article. Prenatal inflammatory stimulation causes NF-κB dyshomeostasis (IκBα/β imbalance) at early life in conduit artery, which in turn reprograms rein-angiotensin-system (RAS) in local conduit artery. RAS over-activity also gives a positive feedback to NF-κB activation. This positive loop drives conduit artery dysfunction and finally promotes hypertension development.

**Table 1:** Primers for realtime RT-PCR.

Gene symbol	Forward primer (5′–3′)	Reverse primer (5′–3′)
*p65*	CACCAAAGACCCACCTCACC	CCGCATTCAAGTCATAGTCCC
*IκBα*	CAACAGTCTGAACTCGCCACC	TCACCATCTGCTCGTAATCCTC
*IκBβ*	GCAGTGACAGCGACAGTGACAAC	TTGGCTCCTGCGACTGTGAAC
*IKKα*	GCAGGGAAAGAGGCAGAAAGA	TGTCAGAGGATGTTCACGGTCT
*IKKβ*	GGTCATCTAATGTCCCAGCCTTC	CTCCATCTGTAACCAGCTCCAGTC
*IKKγ*	CCAGGCGGACATCTACAAGG	GCCAAATGAAAGGAGTGGTGAGC
*TNF-α*	CAAGGCTGCCCCGACTATGTGC	TTGATGGCGGAGAGGAGGCTGAC
*IL-6*	CTTCCAGCCAGTTGCCTTCTTG	GTCTGTTGTGGGTGGTATCCTC
*ACE*	GCGTCAACTTCCTGGGTATGTA	GAGGCTGTGATGGTTATGGATG
*AT1R*	CCCTACCCTCTACAGCATCATCT	GGCGAGATTGAGAAGAAAGACG
*AT2R*	CTCTGACCTGGATGGGTATCAT	GGGAACTCTAAACACACTACGG
*β-actin*	GACGTTGACATCCGTAAAGACC	TAGGA GCCAGGGCAGTAATCT
